# Remote-Sensing Applications for Environmental Health Research

**DOI:** 10.1289/ehp.122-A268

**Published:** 2014-10-01

**Authors:** Nate Seltenrich

**Affiliations:** Nate Seltenrich covers science and the environment from Petaluma, CA. His work has appeared in *High Country News*, *Sierra*, *Yale Environment 360*, *Earth Island Journal,* and other regional and national publications.

More than 1,000 manmade satellites currently orbit our planet.^1^ Some are near the edge of the Earth’s atmosphere just a few hundred kilometers up. Others are tens of thousands of kilometers above us.^2^ They aid in communication, navigation, defense, and science. A small number^3^^,^^4^ play a critical and quickly expanding role: monitoring the Earth’s surface and atmosphere to track environmental conditions that are intimately tied to human health.

**Figure:**
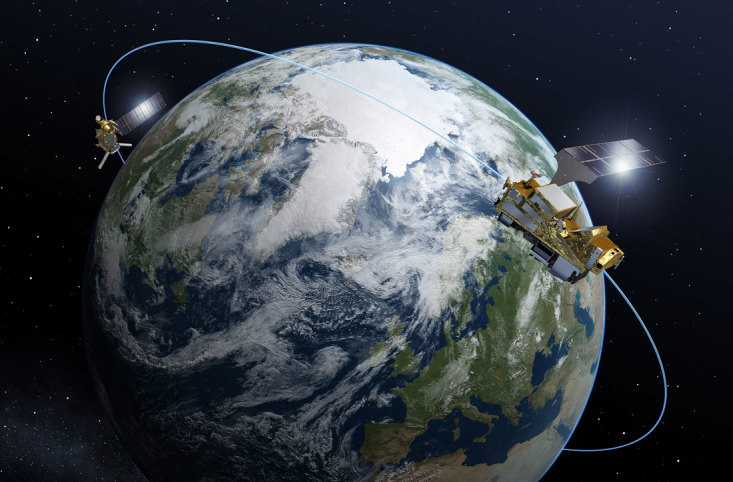
A number of new Earth-observing missions are planned for the next decade, including Sentinel-5 aboard the European Space Agency’s MetOp Second Generation satellites (pictured).^48^ In the meantime researchers are finding new uses for the satellite data currently available. © P. Carril/ESA

Researchers and government agencies worldwide already use satellite data to monitor air pollutants, infectious disease epidemics, harmful algal blooms (HABs), climate change, and more. But as current research indicates, that’s only the beginning of what we can do with the technology, broadly referred to as “remote sensing.” In the coming years, new satellites will offer higher-resolution imagery in conjunction with more robust and precise algorithms to process the data they deliver. As a result, researchers expect to dramatically expand their ability to view and understand Earth’s land, water, and air, from its remotest ocean waters to its largest cities.

The National Aeronautics and Space Administration (NASA) launched its first satellite in 1958,^5^ and TIROS-1, the country’s first meteorological satellite, came 2 years later.^6^ Within a few decades members of the epidemiological and public health communities began actively looking at satellite data, says John Haynes, program manager of the NASA Applied Sciences Health and Air Quality Applications Program. In recent years interest in remote-sensing data has soared, with newer avenues being developed and fine-tuned, including air-quality measurements and vector-borne disease projections. “There’s really been a paradigm shift in the use of remote sensing for public health issues,” Haynes says. “Every year there seems to be more and more interest.”

Indeed, by March 2015 NASA will have launched 6 Earth-observing missions in 12 months,^7^ more than in any year in at least a decade.^8^ New launches include a “global precipitation observatory” that will make frequent global measurements of rain and snowfall, plus one satellite designed to measure soil moisture and another that will measure how carbon moves through the Earth’s atmosphere, land, and oceans. In addition, the International Space Station will receive three new instruments, one that will observe how winds behave around the world, one that will measure clouds and aerosols (particles suspended in the atmosphere)—two variables that remain difficult to predict in climate-change models—and one that will take global, long-term measurements of key components of the Earth’s atmosphere, including aerosols and ozone.^9^ The momentum will carry through at least the next 8 or so years, with NASA and other space agencies in Europe and Asia planning to launch new satellites that will provide even higher-resolution snapshots of the Earth.

Along with technological and scientific advances, a third development is leading to new and improved applications of satellite data: NASA and the National Oceanic and Atmospheric Administration (NOAA) have made their satellite data available free of charge, Haynes says, while the European Space Agency (ESA) has reduced prices and promised to provide free access to data from its next generation of instruments.

“More people use the data, and you get more out of it than when you try to restrict it,” says Raphael Kudela, an oceanographer at the University of California, Santa Cruz, who uses satellite imagery to study HABs. This free sharing of data has been instrumental in his field, allowing researchers at institutions around the world to study HABs from above and to improve systems to track and predict them.

## Tracking HABs

HABs are a growing global concern due to increases in aquaculture activity (which both contributes to and is impacted by blooms), widespread runoff of nutrient-rich fertilizer and sewage into coastal waters, transport of HAB species via ship ballast water, and climate change, which may expand the ranges of some marine species and increase the size and frequency of freshwater blooms.^10^^,^^11^ As a result of heightened awareness and improved detection of HABs thanks to both satellites and water-based sensors, reporting of events has increased in recent years.^12^

HAB research was used as a major justification for some instrument launches, says Don Anderson, a senior scientist and the HAB program lead at the Woods Hole Oceanographic Institution. Research assistant professor Tim Moore of the University of New Hampshire says that through remote sensing, not only can we collect observations of hard-to-reach places, but we’re also able to do it on a continual basis: a double benefit. “That’s why remote sensing is such an appealing platform,” he says.

However, researchers have also come to understand that remote sensing can’t operate alone in HAB research and is best used in conjunction with *in situ* sensors and traditional water sampling. “Now we’re in a much more realistic phase where people understand the limitations and understand the strengths, and they’re much more careful about what is promised or projected,” Anderson says. “It’s a much healthier environment in that way.”

Satellite imagery cannot detect low-biomass blooms such as *Alexandrium fundyense*, a highly toxic dinoflagellate common along the New England coast.^13^ The algae’s deadly toxins accumulate in shellfish during seasonal blooms that sometimes are not visible from feet away, let alone from space, because of their low cell concentrations. “If something doesn’t absorb or scatter light in a significant way, it can’t be picked up with remote sensing,” says Richard Stumpf, a NOAA researcher who leads the agency’s effort to develop regular HAB forecasts.

Remote sensing also falls short when it comes to scanning small freshwater bodies for toxic cyanobacteria because existing satellites do not offer sufficiently high resolution. At least 3 contiguous pixels of imagery are necessary to convey an accurate sense of surface conditions, says Stumpf.

A third shortcoming is that satellites do not always provide the “revisiting frequency” necessary for optimal bloom detection and tracking. In other words, because their sensors are not constantly trained on any one patch of water, and because the pictures they do take may be obscured (for instance, by clouds), researchers may in some cases be limited to just one good image per week, says Moore.

Still, in many cases across the United States and around the world, remote sensing is a critical tool for monitoring HABs, offering a holistic, big-picture view and more spatial context than is available using discrete surface sensors, Kudela says. NOAA relies on satellite imagery to develop its free weekly forecasts for the Gulf of Mexico, where toxic *Karenia brevis* blooms dominate other algal species and concentrate on the ocean’s surface during the daytime; and for Lake Erie, where *Microcystis aeruginosa* blooms float to the surface, forming a bright, readily visible scum, Stumpf says. *Microcystis* threatens the drinking water of millions of people around Lake Erie each summer^14^ and in August 2014 shut down the water supply in Toledo, Ohio.^15^ NOAA forecasts had predicted a significant bloom much like the one that affected Toledo.^16^

**Figure:**
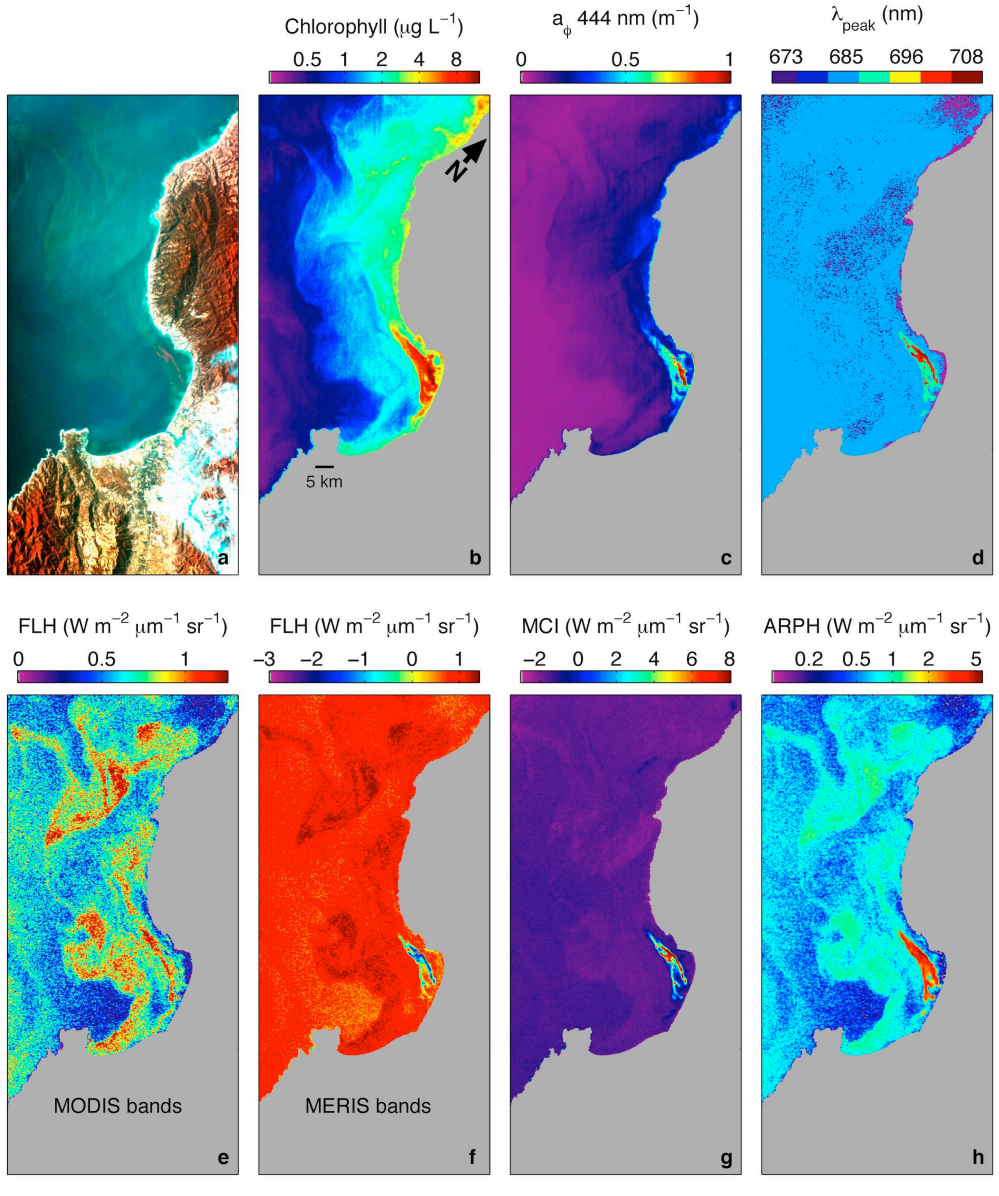
This image of the Monterey Bay region, off central California, was acquired from the International Space Station by the Hyperspectral Imager for the Coastal Ocean (HICO) on 6 November 2012.^53^ The high spatial and spectral resolutions of HICO data permit detection of different types of phytoplankton populations, some of which can form small, densely concentrated patches. Researchers examined how different algorithms characterized the different populations, roughly similar to the way a photo, an X ray, and an MRI reveal different aspects of a medical issue. The image in panel h resulted from an algorithm designed to consistently characterize phytoplankton optical signal across the full range of bloom types and biomass levels, offering a glimpse of what may be possible in the future with the next-generation remote sensing. Image: Ryan et al. (2014)^53^

ESA’s Sentinel-3 mission, set to launch in 2015,^17^ will address the problem of low resolution with an instrument allowing researchers to monitor blooms in lakes as small as 1 km across, says Stumpf. Once Sentinel-3 is active, Kudela notes, “we can take methods that we know work and start to apply them to smaller water bodies almost immediately.”

In anticipation of higher-resolution sensors on the horizon, current research is focused on improving and specializing quantitative algorithms to make better use of the imagery collected by satellites. Moore is developing new algorithms that can better assess the presence of *Microcystis* in Lake Erie and other freshwater bodies by capitalizing on the organism’s ability to absorb and scatter light.^18^ “If you have a good understanding of the optical properties of a species, then you have the potential of developing an algorithm specific to that species,” he says. “The ultimate goal is to detect when a HAB is developing.”

Meanwhile on the West Coast, assistant professor Angelicque White at Oregon State University is using remote sensing to identify predictors of HABs off the Oregon coast, assess risk periods for these events, and develop satellite-detectable proxies of bloom biomass such as sea surface temperature. Yet no matter how refined these tools become, she says, the measurements will never be able to stand alone in a functioning early-warning system. “Remote sensing is a relatively powerful tool for HAB monitoring,” White says, “but it has to be married to some sort of monitoring program.”

## Watching Infectious Diseases

Satellite data also exhibit both shortcomings and great promise in projecting outbreaks of infectious diseases. Researchers have found that environmental factors bear strong relationships to the spread of diseases such as malaria^19^^,^^20^ and meningitis,^21^ among many others. They are now employing satellites to track those factors in order to predict and assess the risk of disease outbreaks, which in turn affects management decisions such as distribution of vaccines.

Yet, much as for HABs, satellite imagery does not provide a complete or perfectly reliable picture of what’s happening on the surface and should be verified by and blended with ground-sensed data, says Madeline Thomson, a senior research scientist with the International Research Institute for Climate and Society (IRI) at Columbia University. For example, the bright surface reflectance in semi-arid areas makes it difficult for satellites to “see” dust there, says Carlos Pérez García-Pando, an associate research scientist at NASA’s Goddard Institute for Space Studies and Columbia University.

Scientists are just beginning to develop hands-on tools rooted in remote sensing such as online maps that help government agencies and health organizations get in front of disease outbreaks. “Abilities of remote sensing to identify particular land-cover types, to monitor changes in vegetation, to provide estimates of rainfall, etc., really go back to the 1980s,” Thomson says. “But for a researcher, our ability to transform that information into something really practical has taken a long time in getting there. I think we’re just getting there now.”

The science of using satellite data to predict outbreaks is most developed around malaria, which has been prioritized because of its global burden, according to Thomson, who has worked in the field for 20 years. Rain is the disease’s primary environmental trigger, as it activates the life cycle of the mosquito by creating breeding sites.^22^ Yet in higher-elevation areas such as the highlands of Kenya, temperature may become the dominant variable,^23^ because in cooler regions, moderate increases in temperature are associated with dramatically increased risk of malaria transmission.^24^ By tracking precipitation and temperature levels with satellites, scientists can provide 2- to 3-month forecasts of malaria epidemics, says Pietro Ceccato, a research scientist and the Environmental Monitoring Program lead at the IRI.

The IRI provides free satellite images and maps displaying malaria risk factors via an extensive online data library.^25^^,^^26^ The organization has developed interfaces that allow nonexperts to visualize different environmental factors over varying time series, as well as to integrate epidemiological data.^27^

“I’ve been working with the ministries of health and the ministries of agriculture [of various countries], and they do use the tool,” Ceccato says. “It’s easy to map those conditions, and they use that information to make decisions.”

The IRI has developed similar maps and predictive tools for meningococcal meningitis outbreaks in Africa’s “meningitis belt,” which extends from Senegal to Ethiopia across the continent’s midsection. More than 800,000 cases of the disease were reported in this region between 1996 and 2010, with 10% being fatal.^28^

The polysaccharide vaccines for meningitis used in the region typically provide immunity for only 2 to 3 years. Therefore, for economic and logistic reasons, the strategy has been reactive vaccination, says Pérez García-Pando; when the number of cases in a certain district reaches a threshold, vaccinations are ordered. But sometimes the response comes too late, so scientists have sought to use what they know about environmental factors in the disease’s transmission to develop forecasts, which they hope will dramatically improve the effectiveness of vaccine distribution.

Meningitis outbreaks in this region of the world have been tied to dry weather and elevated dust levels.^29^ Scientists don’t fully understand the nature of the relationship, but there are 2 leading hypotheses, says Pérez García-Pando: first, dust particles in the air irritate the mucous membranes and facilitate entry of meningococcal bacteria into the bloodstream; and second, the high iron content in dust from this region serves as a nutrient for the bacteria, promoting colonization and increasing transmission and infection.

No matter the mechanism, researchers have observed that just as dust storms in the dry season facilitate the transmission of the disease, high-humidity events tamp down and often put an end to epidemics, says Thomson. Monitoring humidity levels could thus prove a valuable tool to health ministries who need to know when and where to provide vaccines.

A long-term study of the link between weather and meningitis by the Colorado-based University Corporation for Atmospheric Research (UCAR) has determined that the best predictor of meningitis activity is the relative humidity 2 weeks prior.^30^ “It’s a very robust result, and we see it again and again in the data,” says Rajul Pandya, director of the UCAR Office of Education and Research, and coordinator of its meningitis project. “It corresponds with what the people who live there know—that meningitis is only a problem in hot, dusty times.” UCAR researchers have concluded that if humidity is expected to exceed 40% in a particular area two weeks hence, health managers should consider allocating the vaccine elsewhere, says Pandya.

## Monitoring Pollutants

Satellites can even be used to detect more ethereal threats, such as nitrogen dioxide (NO_2_) and fine particles (PM_2.5_) suspended near the Earth’s surface. Recent advances have shed new light on our understanding of global air pollutant concentrations, with significant implications for public health. Even short-term exposures to elevated NO_2_ levels may trigger a range of adverse respiratory effects, in both sick and healthy people,^31^ whereas particulate pollution impacts both the cardiovascular and respiratory systems and is associated with premature death in people with heart or lung disease.^32^

To detect NO_2_, scientists can use advanced sensors orbiting the Earth, including the Ozone Monitoring Instrument (OMI) aboard NASA’s Aura satellite^33^ and the Global Ozone Monitoring Experiment 2 (GOME-2) instruments on ESA’s MetOp-A satellite.^34^

And for PM_2.5_, available instruments include the Moderate Resolution Imaging Spectroradiometer (MODIS) on NASA’s Aqua and Terra satellites^35^ and the Multi-angle Imaging SpectroRadiometer (MISR) that flies on Terra.^36^ In both cases, researchers scan a vertical column of air between the satellite and the Earth’s surface—like drilling an ice core, but without actually extracting anything—and then use models to estimate the proportion of the overall concentration that exists near the Earth’s surface, typically within 100 m.

A team at Dalhousie University in Halifax, Nova Scotia, has recently completed groundbreaking work on both fronts. In particular, their 2010 report in *EHP* was the first to show a long-term global map of PM_2.5_ distribution, in this case representing a 6-year average.^37^ The map offers an overhead view of the planet’s landforms color-coded to display a range of PM_2.5_ concentrations. A broad swath extending from Africa’s Sahara Desert to eastern Asia appears orange, red, and deep red, representing very high levels, while much of central Europe is colored yellow, representing medium levels, and the vast majority of the rest of the planet, including most of the United States, is colored light and dark blue, representing low levels. (NASA and EPA partnered earlier this year to integrate satellite data into similar maps for the United States and part of Canada.^38^)

“The sheer scale of this PM_2.5_ enhancement wasn’t fully appreciated prior to the satellite observations,” says study coauthor Randall Martin, a professor of atmospheric composition at Dalhousie University. “The satellites that we used have been around for almost fifteen years, and it took ten years since their launch to create this paper that contains the map that you see today. That emphasizes that there was a lot of effort by a lot of scientists that made this all possible.”

**Figure:**
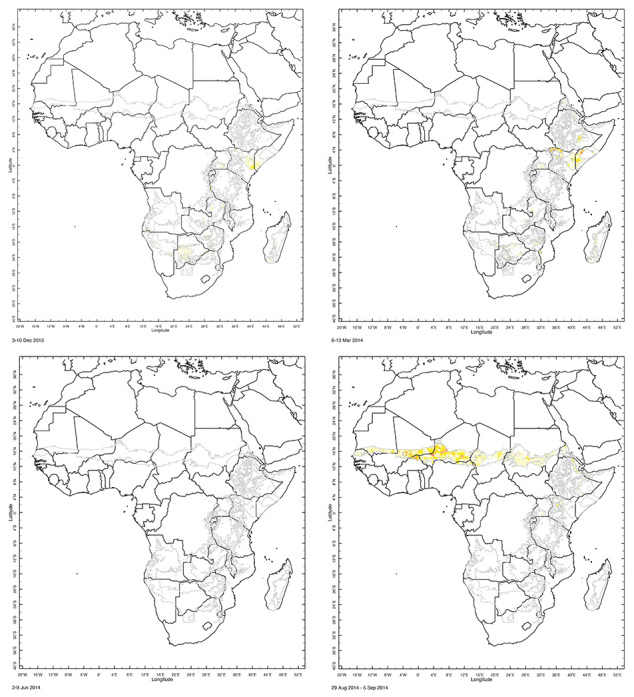
Researchers at Columbia’s International Research Institute for Climate and Society use precipitation and temperature data collected remotely to forecast outbreaks of malaria and meningitis in Africa. Malaria outbreaks are tied not only to rainfall but also, in some areas, to temperature. Satellite tracking enables researchers to forecast epidemics 2 to 3 months in advance. Image: IRI^26^

**Figure:**
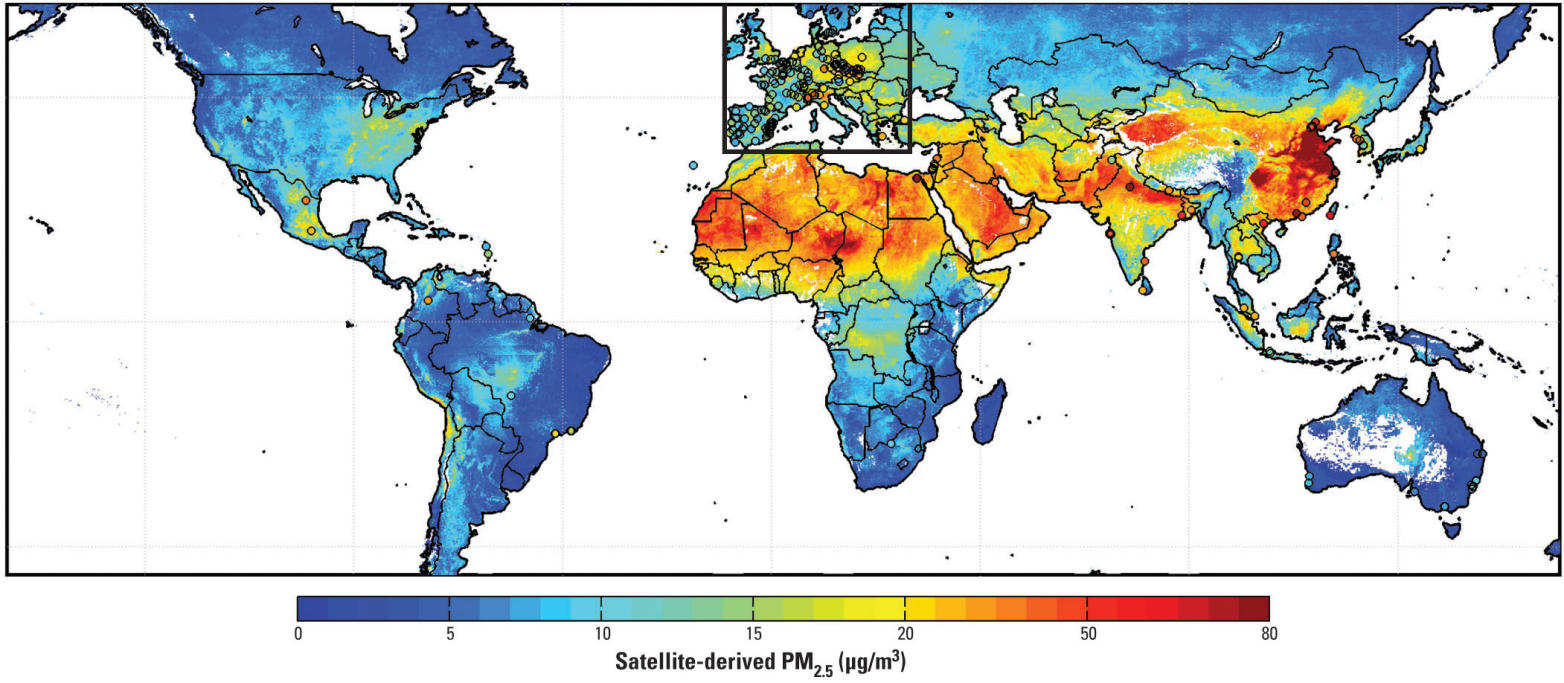
Groundbreaking research by Aaron van Donkelaar and colleagues produced the first long-term global map of PM_2.5_ distribution. Using satellite data the team was able to provide estimates of air quality for regions without ground-level sampling. According to the authors’ estimates, 80% of the global population lives in places where concentrations of PM_2.5_ exceed the World Health Organization (WHO) air-quality guideline of 10 µg/m^3^.^37^ Image: van Donkelaar et al. (2014)^37^

Prior research focused on cities where ground-based sensors were available, which excluded most rural areas. “There really is a large amount of demand [for these data], because it’s one of the few relatively consistent global data sets,” says lead author Aaron van Donkelaar, a postdoctoral fellow in Dalhousie’s Department of Physics and Atmospheric Science. “Global air quality is something that I think needs to be addressed. I’m proud of the contribution this data has made to understanding air quality in general and the disparity in air quality that exists around the world.” A followup study is currently under review at *EHP*.

The Boston-based nonprofit Health Effects Institute is among those employing the team’s data, says Aaron Cohen, a principal scientist at the institute who manages an epidemiological program on the health effects of air pollution.^39^ “Their estimates were key because they give us complete global coverage at a fairly fine spatial scale. We are able to include the whole globe for the first time,” he says. “They’ve in many ways transformed the kind of health effects studies we can do, in terms of their size and their scope, once you don’t have to depend [solely] on the presence of ground-level monitors.”

In 2008 some of the same authors published the world’s first assessment of ground-level NO_2_ concentrations using satellite data.^40^ The study focused on North America as a proof of concept, says van Donkelaar, and in the years since, additional studies have addressed other regions.^41^^,^^42^ He notes that readings may offer insight into specific sources of air pollution as opposed to a more averaged distribution in the atmosphere.

Lok Lamsal, lead author of the NO_2_ study, is now a research scientist at the Goddard Space Flight Center, where his focus remains on satellite-derived measurements of NO_2_ concentrations. Researchers have more confidence in remotely sensed NO_2_ data than they do for some other pollutants such as sulfur dioxide and formaldehyde, he says. But NO_2_ measurements are currently hampered by the relatively low resolution of the images from OMI, which is the primary instrument that provides them. Furthermore, OMI has experienced a malfunction that renders half the pixels it provides unusable, Lamsal says.

OMI’s resolution of about 10 to 20 km^2^ will be improved upon by the Tropospheric Monitoring Instrument (TROPOMI) part of ESA’s Sentinel-5P mission, set to launch in 2016.^43^ NASA has also proposed to launch a new satellite instrument, Tropospheric Emissions: Monitoring of Pollution (TEMPO), that will be able to measure NO_2_ concentrations in higher resolution on an hourly basis, offering greater levels of spatial and temporal resolution.^44^

“There will be much improved data quality that we can look at in the next couple of years,” Lamsal says. “There are certainly huge improvements in the last ten years regarding information on nitrogen dioxide, but that will improve [even more] in the next couple years.”

In the meantime, he and other NASA researchers continue to refine the models and algorithms that convert available satellite data into useful products, as well as assist air-quality researchers and managers in applying them through the agency’s Air Quality Applied Sciences Team.^45^ Yet even as new algorithms and instruments improve researchers’ ability to remotely sense atmospheric NO_2_, Martin believes ground sensors will remain essential counterparts, offering both high spatial resolution and vertical profile information.

## The Next Generation

Most of the instruments that make environmental monitoring possible live aboard satellites in low-Earth orbit, meaning they’re at an altitude between 160 km (with an orbital period of around 88 minutes) and 2,000 km (with an orbital period just over 2 hours). NASA currently operates 17 such satellites, Haynes says, which serve human health research and management in many ways, including through natural-disaster forecasting, mitigation, and response; ecological forecasting, including for HABs; supporting air-quality management and policy; and assessing water availability and quality.^46^ NOAA also operates a number of Earth science satellites with similar objectives.^47^

These will soon be joined, or replaced, by next-generation environmental sensors that can support all the existing avenues of research as well as open up new ones, initiating a new era in satellite-based environmental health research. For instance, TROPOMI, developed by ESA in partnership with the Netherlands, will make daily observations of a broad range of atmospheric compounds of health concern: ozone, NO_2_, sulfur dioxide, carbon monoxide, methane, formaldehyde, and aerosols. It can detect everything that its predecessor OMI can, plus more that it can’t, all at a higher resolution.

Sentinel-3’s Ocean Land Colour Instrument, also from ESA, will prove a boon to HAB researchers with its higher resolution. (Sentinel-3 will be followed by 3 other missions by 2020, all performing Earth-observation missions.^48^) And NASA’s Pre-Aerosol, Clouds, and Ocean Ecosystem mission, scheduled for launch in 2019 or 2020, will offer hyperspectral imaging of the ocean surface (which involves collecting data simultaneously in a large number of narrow adjacent-wavelength bands) as well as monitor atmospheric chemistry.^49^

Also planned for the next decade is NASA’s Geostationary Coastal and Air Pollution Events mission. This geostationary satellite positioned to view North and South America and adjacent oceans will measure tropospheric trace gases and aerosols as well as coastal ocean phytoplankton, water quality, and biogeochemistry.^50^ And the Hyperspectral Infrared Imager, developed by NASA with the California Institute of Technology and scheduled for launch in 2022, will collect data designed to monitor ecological and environmental health factors such vegetation cover, drought, and wildfires.^51^

Still more instruments will be launched over Asia. Many Asian nations—including China, Japan, India, and South Korea—are members of the international Group on Earth Observations (GEO), formed in 2003 to improve access to Earth-science data,^52^ but as a whole they haven’t committed to making their data available in the same way that NASA, NOAA, and ESA have, Kudela says.

While not infallible, the new satellites are brimming with promise. They will help scientists identify microscopic algal cells from hundreds of kilometers away, provide health-care workers with life-saving information about disease epidemics, and remotely monitor the air being breathed by residents of some of the planet’s most far-flung locales—all this within six decades of man’s first foray into space.
