# Erratum: “Remote-Sensing Applications for Environmental Health”

**DOI:** 10.1289/ehp.122-a296

**Published:** 2014-11-01

The October 2014 News article “Remote-Sensing Applications for Environmental Health” [Environ Health Perspect 122:A268–A275 (2014); http://dx.doi.org/10.1289/ehp.122-A268] incorrectly stated that the Boston-based nonprofit Health Effects Institute is “looking forward to a followup study currently under review at *EHP*.” The mention of this study should have appeared in an earlier paragraph.

*EHP* regrets the error.

